# Non-*Helicobacter pylori Helicobacter* (NHPH) positive gastric cancer

**DOI:** 10.1038/s41598-022-08962-y

**Published:** 2022-03-21

**Authors:** Tomohiko Yasuda, Hyun Seok Lee, Su Youn Nam, Hiroto Katoh, Yuko Ishibashi, Somay Yamagata Murayama, Hidenori Matsui, Hiroki Masuda, Emiko Rimbara, Nobuyuki Sakurazawa, Hideyuki Suzuki, Hiroshi Yoshida, Yasuyuki Seto, Shumpei Ishikawa, Seong Woo Jeon, Masahiko Nakamura, Sachiyo Nomura

**Affiliations:** 1 Department of Gastrointestinal Surgery, Graduate School of Medicine, The University of Tokyo, 7-3-1 Hongo, Bunkyo-ku, Tokyo, 113-8655 Japan; 2 Department of Gastrointestinal Surgery, Nippon Medical School Chiba Hokusoh Hospital, Chiba, Japan; 3 Department of Internal Medicine, School of Medicine, Kyungpook National University, Kyungpook National University Hospital, Daegu, Korea; 4 Department of Preventive Medicine, Graduate School of Medicine, The University of Tokyo, Tokyo, Japan; 5 Department of Breast and Endocrine Surgery, Graduate School of Medicine, University of Tokyo, Tokyo, Japan; 6 Department of Chemotherapy and Mycoses, National Institute of Infectious Diseases, Tokyo, Japan; 7 Omura Satoshi Memorial Institute, Kitasato University, Tokyo, Japan; 8 Department of Gastrointestinal and Hepato-Biliary-Pancreatic Surgery, Nippon Medical School, Tokyo, Japan; 9 Department of Bacteriology II, National Institute of Infectious Diseases, Tokyo, Japan

**Keywords:** Cancer, Microbiology, Molecular biology, Gastroenterology, Medical research, Pathogenesis

## Abstract

Genetic analysis and culturing techniques for gastric non-*Helicobacter pylori Helicobacter* (NHPH) are progressing. NHPH is reported to accompany nodular gastritis, gastric MALT lymphoma, and mild gastritis. However, only a few gastric cancer cases infected by NHPH have been reported. PCR analysis specific for NHPH and *H. pylori* was performed for DNA from gastric mucosa of 282 Korean gastric cancer patients, who were treated with endoscopic submucosal dissection. For more precise strain detection of NHPH, NHPH-positive mucosa was stained by immunohistochemistry specific for *Helicobacter suis*. The Cancer Genome Atlas (TCGA) classification was analyzed for these 3 gastric cancer sub-groups by in situ hybridization and immunohistochemistry. Among 281 patients, 3 patients (1.1%) were positive for NHPH. One patient (Patient 1) was also positive for *H. pylori* by PCR, another patient (Patient 3) was positive for serum IgG for *H. pylori*, and the other patient (Patient 2) had no evidence for *H. pylori* infection. Gastric mucosa of Patients 2 and 3 were positive for *H. suis* staining. All three NHPH-positive gastric cancers were located in the antrum, and belonged to the Chromosomal Instability Type of TCGA classification. Gastric NHPH can be a cause of gastric cancer, although likely with lower pathogenesis than *H. pylori*.

## Introduction

In recent years, the importance of non-*Helicobacter pylori Helicobacter* (NHPH) is increasing due to advances in culturing techniques and genetic analysis for NHPH^1^. Eradication for *Helicobacter pylori* is also contributing to a focus on gastric diseases caused by NHPH. Gastric NHPH was originally reported as *Gastrospirillum hominis* in 1900 and subsequently renamed *H. heilmannii*^2–4^*,* having a larger body (5–6 μm × 0.5–0.6 μm) than *H. pylori*, with flagella on both sides. Gastric NHPH is reported to live in livestock and pets, and is found in 0.2–6% of gastric biopsies^5^. Gastric NHPH is associated with mild gastritis, nodular gastritis, and gastric mucosa associated lymphoid tissue (MALT) lymphoma^6–8^. However, there are only a few reports of NHPH associated with gastric cancer^9–11^.

In this study, we investigated the presence of NHPH in gastric mucosa in 281 Korean early gastric cancer patients, treated with endoscopic submucosal dissection (ESD). We found 3 gastric cancer patients with NHPH infection by NHPH-specific PCR. Clinicopathological features of these gastric cancers infected with NHPH will be presented.

## Materials and methods

### Subjects

All 281 samples were collected from the patients with early gastric cancer treated with endoscopic submucosal dissection at the Kyungpook National Univerity Chilgok Hospital in Korea from January 2011 to May 2013. All the cancer was confirmed to be well-differentiated adenocarcinoma by biopsy prior to endoscopic submucosal dissection. We also collected blood serum samples from these patients before endoscopic treatment. All patients had no history of *H. pylori* eradication. The biopsy specimens for this study were provided by the National Biobank of Korea, Kyungpook National University Chilgok Hospital (KNUCH), which is supported by the Ministry of Health, Welfare and Affairs Korea. All materials derived from the National Biobank of Korea, KNUCH, were obtained under institutional review board-approved protocols. Informed consent was obtained from all subjects and/or their legal guardian. The research was also approved by the Kitasato Institute Hospital Review Board (16059).

The precise description of clinicopathological data for these patients is presented in our previous report^12^.

Gastric mucosal biopsies from all the patients were analyzed by Rapid Urease Test. Anti-*H. pylori* IgG, Pepsinogen I, Pepsinogen II were measured and the patients were classified into A, B, C, and D groups according to ABC method. A group is *Helicobacter pylori* IgG negative and pepsinogen test negative. B group is *Helicobacter pylori* IgG positive and pepsinogen test negative. C group is *Helicobacter pylori* IgG positive and pepsinogen test positive. D group is *Helicobacter pylori* IgG negative and pepsinogen test positive. Anti-*Helicobacter Pylori* IgG level was measured with latex agglutination turbidimetry by SRL Co. (SRL Ltd, Tokyo, Japan). When this level was more than 9.9 U/ml, the infectious status was judged as positive. Serum pepsinogen I and pepsinogen II level were measured by SRL Co. (SRL Ltd, Tokyo, Japan). Lower pepsinogen I/II ratio suggests more severe atrophy and the pepsinogen test was judged as positive when the ratio was less than 3.0 and pepsinogen I < 70 ng/ml.

### DNA extraction and PCR

DNA was extracted from four 10 µm thick paraffin sections for each non-cancerous mucosa surrounding cancer with QIAamp DNA FFPE Tissue Kit (Qiagen Japan, Tokyo). All the extracted DNA was examined by real-time PCR for *H. pylori* and gastric NHPH using iQ™ SYBR Green supermix (Bio-Rad Laboratories). The real time PCR analysis was performed under the conditions of 5 min of pre-incubation at 95 °C, followed by 40 cycles of 30 s at 94 °C, 30 s at 60 °C, and 30 s at 72 °C. A final extension was performed for 7 min at 72 °C. And melting curve was confirmed with a stepwise increase in temperature from 55 to 95 °C in 0.5 °C/5 s increments. For the positive control for PCR, DNA extracted from *H. suis* infected mouse gastric sections was used, and for the negative control for PCR, DNA extracted from human gastric mucosal sections not infected with *Helicobacter* was used. The sequences of primers for *H. pylori*, NHPH, *H. suis*, *H. heilmannii* s.s., *H. bizzozeronii*, *H. felis*, and *H. salomois* are listed in Table 1. The NHPH primers could detect *H. suis*, *H. heilmannii* s.s., *H. bizzozeronii*, *H. felis*, and *H. salomois*^13–16^. For *H. pylori*, the primers for the vacA gene were used. The PCR products for NHPH were purified from agarose gels using QIAquick Gel Extraction Kit (Qiagen Japan, Tokyo), and sequenced by Eurofins Genomics Japan.

**Table 1:** Primers for PCR.

Primer	Target gene	Species	Sequence (5′–3′)	Products (bp)	References
VAC3624F	*vacA*	*H. pylori*	GAGCGAGCTATGGTTATGAC	229	^13^
VAC3853R	*vacA*	*H. pylori*	ACTCCAGCATTCATATAGA		^13^
HeilF	16S rDNA	NHPH	AAGTCGAACGATGAAGCCTA	112	^14^
HeilR	16S rDNA	NHPH	ATTTGGTATTAATCACCATTTC		^14^
T1ureF	*ureA*	*H. suis*	CAAATTTTCCYGATGGAACTA	323	^15^
T1ureR	*ureA*	*H. suis*	GCCGCCYACATCAATYAAATGC		^15^
T2ureF	*ureA*	*H. heilmannii* s. s	AAGTGGGGATTGAAGCGGGC	376	^15^
T2ureR	*ureA*	*H. heilmannii* s. s	CGATCSACTAGAGCGTTGAAA		^15^
UreAF	*ureA*	*H. bizzozeronii*,	CGCTTTGAACCCGGTGAGAAAA	172	^16^
UreAR	*ureA*	*H. bizzozeronii*,	TATCGCAACCGCAATTCACAACA		^16^
UreBF	*ureB*	*H. felis*,	TCCCACTACCGGGGATCGTG	350	^16^
UreBR	*ureB*	*H. felis*,	CAGCGGTTACAATCAAGCCCTCA		^16^
UreABF	*ureAB*	*H. salomonis*,	CTTTGGGTCTGTGCCTGCCTG	219	^16^
UreABR	*ureAB*	*H. salomonis*,	CATCGCGGATAGTCTTACCGCCT		^16^

### Immunohistochemistry and in situ hybridization

We performed immunohistochemical analyses for NHPH-positive gastric cancers with anti-p53, E-cadherin (CDH1), and MLH1 antibodies, as well as in situ hybridization for Epstein–Barr virus-encoded small RNA (EBER), for the Cancer Genome Atlas (TCGA) classification^17,18^.

For the immunohistochemistry, histopathological specimens of the gastric cancers were deparaffinized by immersion in xylene solution (#241-00091, FUJIFILM Wako Pure Chemical Corporation, Japan) for 10 min at room temperature; then, antigen retrieval was performed by autoclave in citric acid buffer (pH = 6.0) (ab64214, abcam, UK) for 5 min at 121 °C. The slides were then immersed in 0.3% H_2_O_2_ (#081-04215, FUJIFILM Wako Pure Chemical Corporation) / methanol (#137-01823, FUJIFILM Wako Pure Chemical Corporation) solution for 10 min at room temperature to remove endogenous peroxidase. Blocking of non-specific reactions was performed by incubating the slides with 2.0% bovine serum albumin (A1470, Sigma Aldrich, MO, USA) / phosphate-buffered saline (PBS) (#045-29795, FUJIFILM Wako Pure Chemical Corporation) solution. Primary antibodies were incubated on the slides overnight at 4℃. The antibodies used were anti-p53 antibody (DO-7) (MA5-12557) at 1/25 dilution, anti-CDH1 antibody (HECD-1) (#13-1700) at 1/100 dilution, and anti-MLH1 antibody (G168-15) (MA1-25669) at 1/25 dilution (all antibodies were from Thermo Fisher Scientific, MA, USA). After washing 3 times with PBS, the antigen–antibody complexes on the slides were visualized using Histostar^(^™^)^ (#8460, MBL, Japan) and DAB Substrate Solution (#8469, MBL). Finally, cell nuclei were stained with hematoxylin (Sakura Finetech, Japan, # 8650).

For the in situ hybridization, EBER PNA Probes/Fluorescein and FITC PNA ISH Detection Kit (Y5200-01 & K5201-11, Agilent technologies, CA, USA) were used according to the manufacture’s protocols.

For immunohistochemistry of *H. suis*, rabbit polyclonal antibody against *H. suis* was made. Briefly, a rabbit was immunized with a peptide expected to be antigenic from the *vacA* paralog reported in a literature^19^. Rabbit IgG was purified from the serum with Protein G affinity column. ELISA results for the specificity of this antibody are shown in Supplemental Fig. S1.

All methods were carried out in accordance with relevant guidelines and regulations.

## Results

### Patients

The characteristics of all 281 gastric cancer patients whose samples were obtained in this study were described in our previous study^12^. Briefly, the patients received endoscopic submucosal dissection for their intestinal-type early gastric cancer. Based on the ABC method, these patients were classified as 22.1% (62/281) in group A, 38.1% (107/281) in group B, 27.0% (76/281) in group C, and 12.8% (36/281) in group D. The positive rate of *H. pylori* IgG was 65.1%. These results are shown in Supplemental Table S1.

### PCR

**Figure 1:**
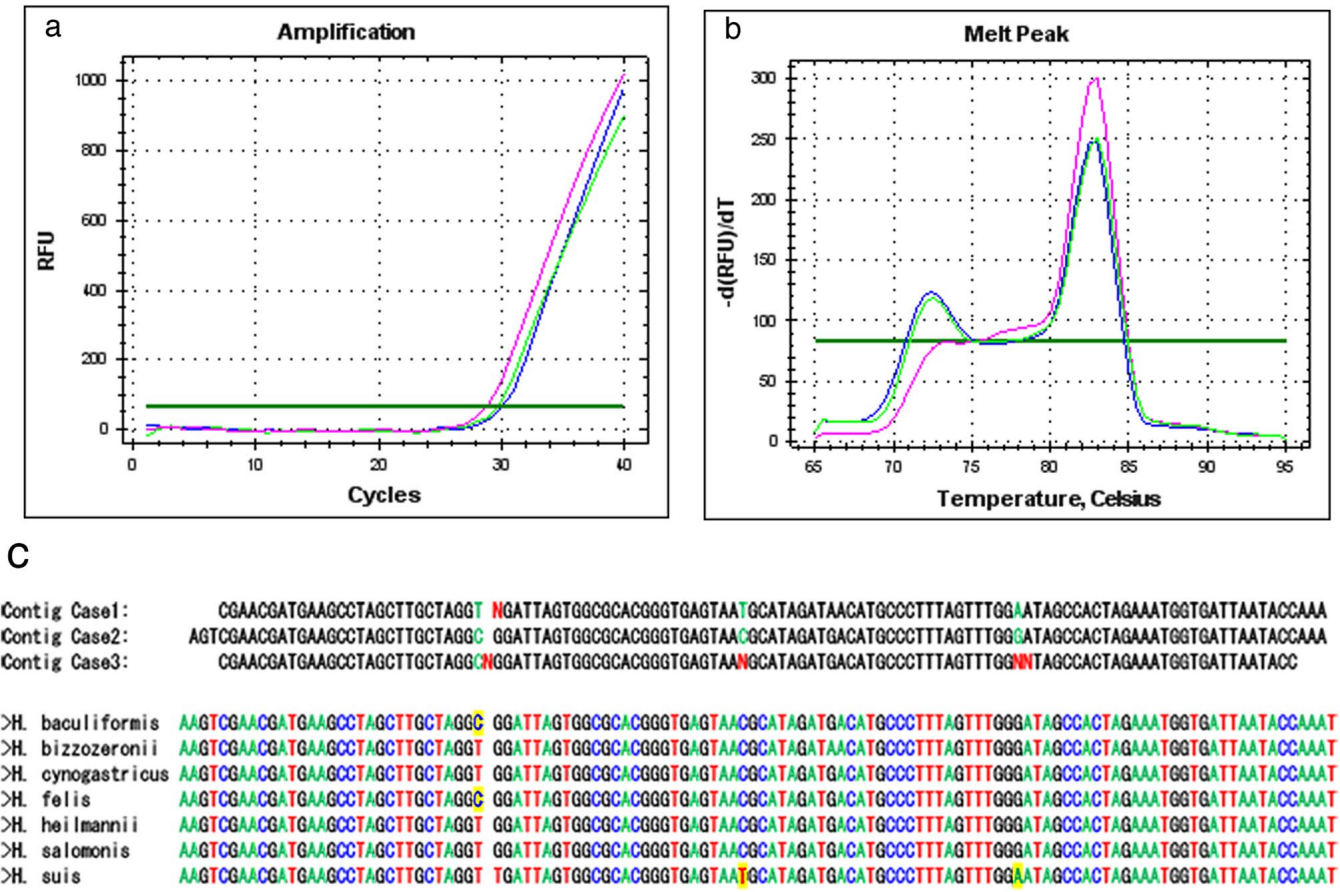
NHPH PCR. (**a**) NHPH PCR amplification curves for the three positive patients are shown. The amplification curves rise around 26 cycles. Three independent experiments showed the similar results. (**b**) Melting curves of these three PCR products are shown, demonstrating specific products. (**c**) Sequence results of PCR products from the three patients aligned with various NHPH strains colonized in stomach.

A 112-bp *ureA* gene fragment specific for NHPH was amplified by PCR from 3 patients out of 281 (Fig. 1). However, gene fragments specific for *H. suis*, *H. bizzozeronii*, *H. felis*, and *H. salomonis* were not amplified by PCR from any of the 3 patients. For *H. suis*, we used two sets of primers for PCR, but gene specific fragments were not amplified, even though the fragments were amplified from DNA extracted from *H. suis* infected mouse gastric mucosa. *H.pylori* specific *vacA* gene was analyzed with PCR for these 3 patients, and 1 patient (Patient 1, 68 year old male) out of 3 patients was positive for *H. pylori*. The sequence results of NHPH PCR amplicon of the 3 patients are shown in Fig. 1C, aligned with reported sequences of *H. baculiformis, H. bizzozeronii, H. cynogastrious, H. felis, H. heilmannii, H. salomonis,* and *H. suis*. There are no reported sequences that match the patient NHPH sequence, and it was also difficult to say the similarity, as the amplicon is too short.

Of the three patients positive for NHPH, two were in group A (Patient 1 and Patient 2) and the other one was in group B (Patient 3).　One patient in group A (Patient 2) was negative for the *H. pylori-*specific *vacA* gene on PCR and may have only had infection with NHPH. The other patient in group A (Patient 1) was positive for the *H. pylori-*specific *vacA* gene by PCR, even though serum *H. pylori* IgG was negative, and had co-infection of NHPH and *H. pylori*. The group B patient (Patient 3) was negative for the *H. pylori*-specific *vacA* gene on PCR and had no medical history of eradication therapy. Table 2 describes the main findings for the 3 patients positive for NHPH.

**Table 2:** NHPH related characteristics of the 3 patients.

	Case 1	Case 2	Case 3
Age	68	64	75
Sex	M	M	M
Location of cancer	Antrum	Antrum	Antrum
Depth of cancer	M*	M	M
Macroscopic type	0-IIc	0-IIb	0-IIc
Histological type	Intestinal	Intestinal	Intestinal
PCR NHPH	Positive	Positive	Positive
PCR *H. pylori*	Positive	Negative	Negative
PCR *H. suis*, *H. heilmannii* s.s., *H. bizzozeronii*, *H. felis*, and *H. salomois*	Negative	Negative	Negative
Kimura-Takemoto classification	C-III	C-II	O-III
*Hp* IgG (U/ml)	4	< 3	39
Pepsinogen I (ng/ml)	65.8	93.1	106.3
Pepsinogen II (ng/ml)	15.5	14.6	24.3
Pepsinogen I/II	4.3	6.4	4.4
ABCD Classification	A	A	B
Rapid Urease Test	Negative	Negative	Negative
TCGA Classification	CIN**	CIN	CIN

*Depth M is confining to mucosa. **CIN is Clomosomal Instability type.

### NHPH immunohistochemistry

**Figure 2:**
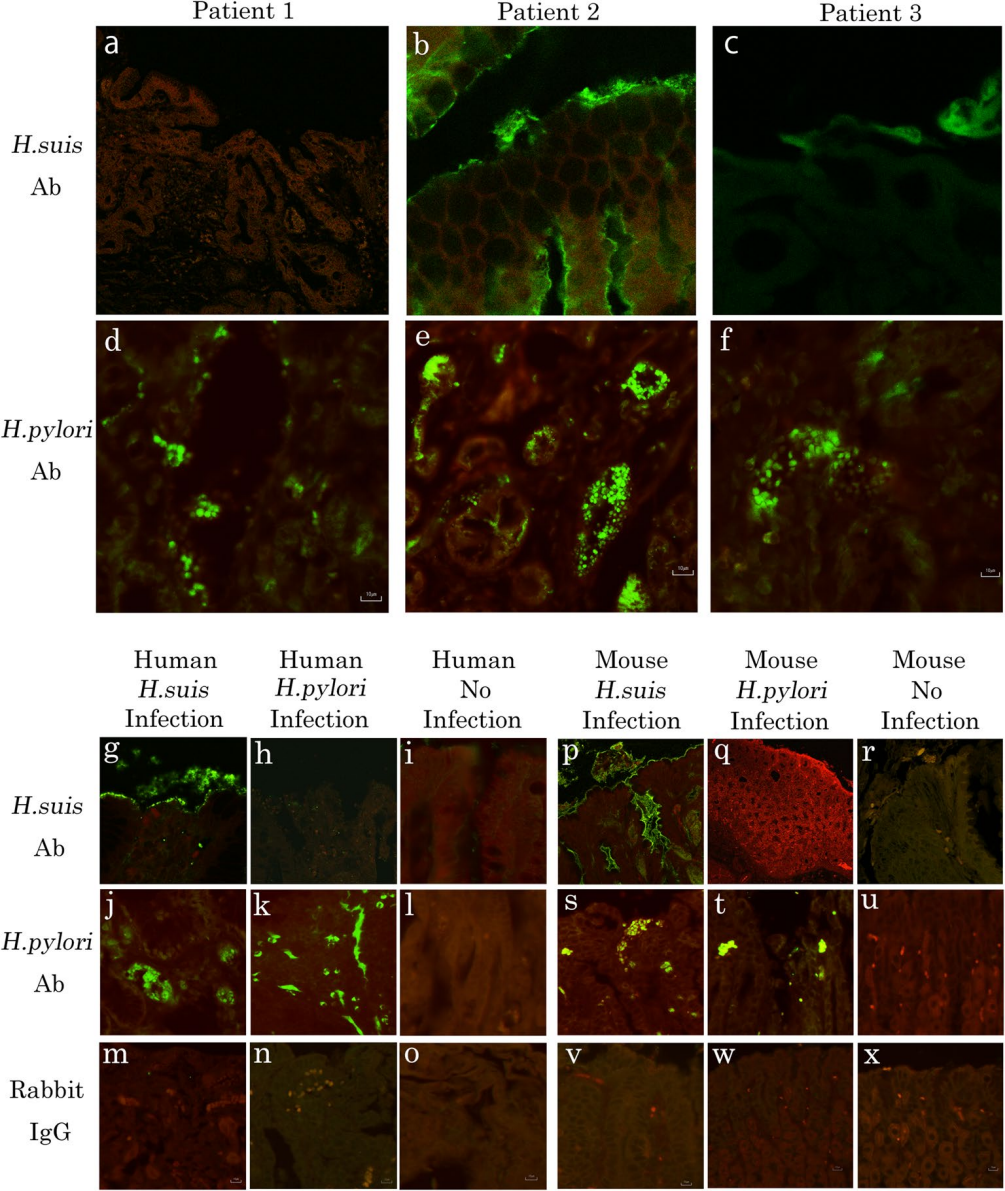
Immunofluorescent staining for *Helicobacter suis* (*H. suis*) HsvA and *H. pylori*. (**a**–**c**) Immunofluorescence staining of *H. suis* for the three NHPH positive patients’ gastric mucosa is shown. (**a**) Gastric mucosa of Patient 1 was negative for *H. suis* staining. (× 476). (**b**) Gastric mucosa of Patient 2 was strongly positive for *H. suis*. *H. suis* was located not only to surface mucosa, but also to deep in the glands. (× 476). (**c**) Surface mucous cells in glands of Patient 3 was also positive for *H. suis*. (× 1890). (**d**–**f**) Immunofluorescence staining with *H. pylori* antibody which reacts also with NHPH. (**g**–**o**) Positive control and negative control of each antibody for human gastric mucosa. (**p**–**x**) Positive control and negative control of each antibody for mouse gastric mucosa.

As PCR for the precise strain of NHPH was negative for the NHPH positive patients, we performed immunohistochemistry for *H. suis*, which is reported to be the most prevalent NHPH for human stomach^19,20^. *H. suis* was strongly positive in Patient 2, weakly positive in Patient 3 and negative in Patient 1 (Fig. 2). Thus, the NHPH in Patients 2 and 3 appeared to be *H. suis*. The positive staining of *H. suis* in Patient 2 was located deep in the gastric glands, as well as in surface epithelium.

### Endoscopy

**Figure 3:**
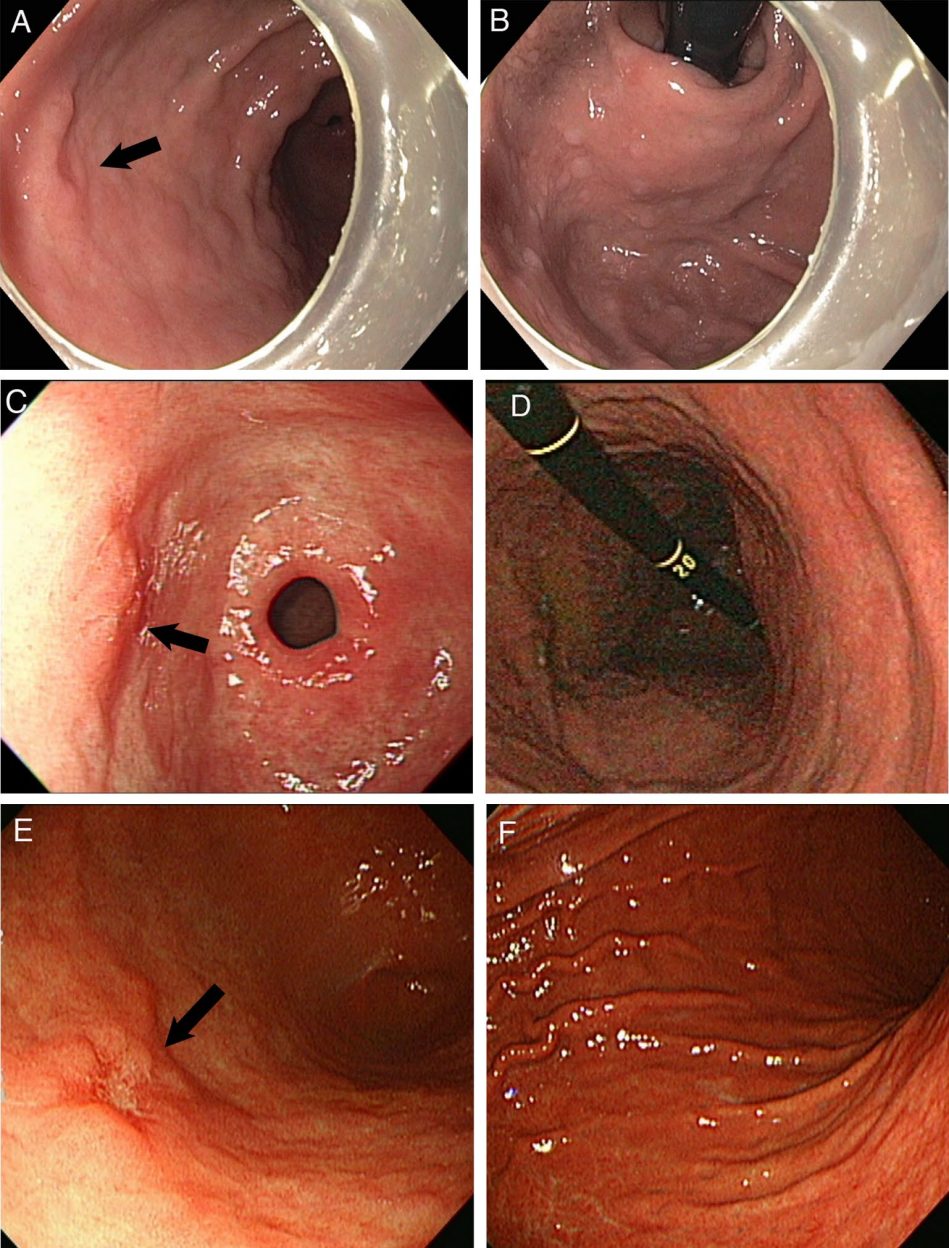
Upper gastrointestinal endoscopic views of the three patients. (Patient 1: A, B, Patient 2: C, D, Patient 3: E, F). All three gastric cancers are located in the anterior wall of antrum. Arrow: Cancer. (**A**) Gastric cancer of Patient 1 is located in the anterior wall of antrum. Macroscopically 0-IIc type superficial depressed type. (**B**) Cardia of Patient 1. Atrophy and gastritis are minimal. Multiple white flat elevations are observed. (**C**) Gastric cancer of Patient 2 is located in the anterior wall of pre-pylorus. Macroscopically 0-IIc type superficial depressed type. The background mucosa shows edema. (**D**) Corpus of Patient 2. The corpus mucosa shows no atrophy and gastritis. (**E**) Gastric cancer of Patient 3 is located in the anterior wall of antrum. Macroscopically 0-IIc type superficial depressed type. The background mucosa shows atrophy. (**F**) Corpus of Patient 3 shows atrophic gastritis with diffuse erythema.

The endoscopic features of gastric cancer and corpus mucosa in these three patients are shown in Fig. 3. Cancer in all three patients was located in the anterior wall of the antrum. The NHPH mono-infected patient (Patient 2) had a flat type (0-IIb) lesion and the other two patients, the *H. pylori* PCR positive patient (Patient 1) and the *H. pylori* IgG positive patient (Patient 3), had a depressed type (0-IIc) lesion.

The atrophic mucosa of Patient 1 and Patient 2 was confined distally and their stomach demonstrated a closed type of Kimura-Takemoto classification in the endoscopic observation. Inflammation was observed in antrum of Patient 2, but the inflammation of corpus mucosa was minimal. The corpus mucosa of Patient 3 was open type, and atrophic gastritis was severe in the corpus compared to other two patients.

### TCGA classification

**Figure 4:**
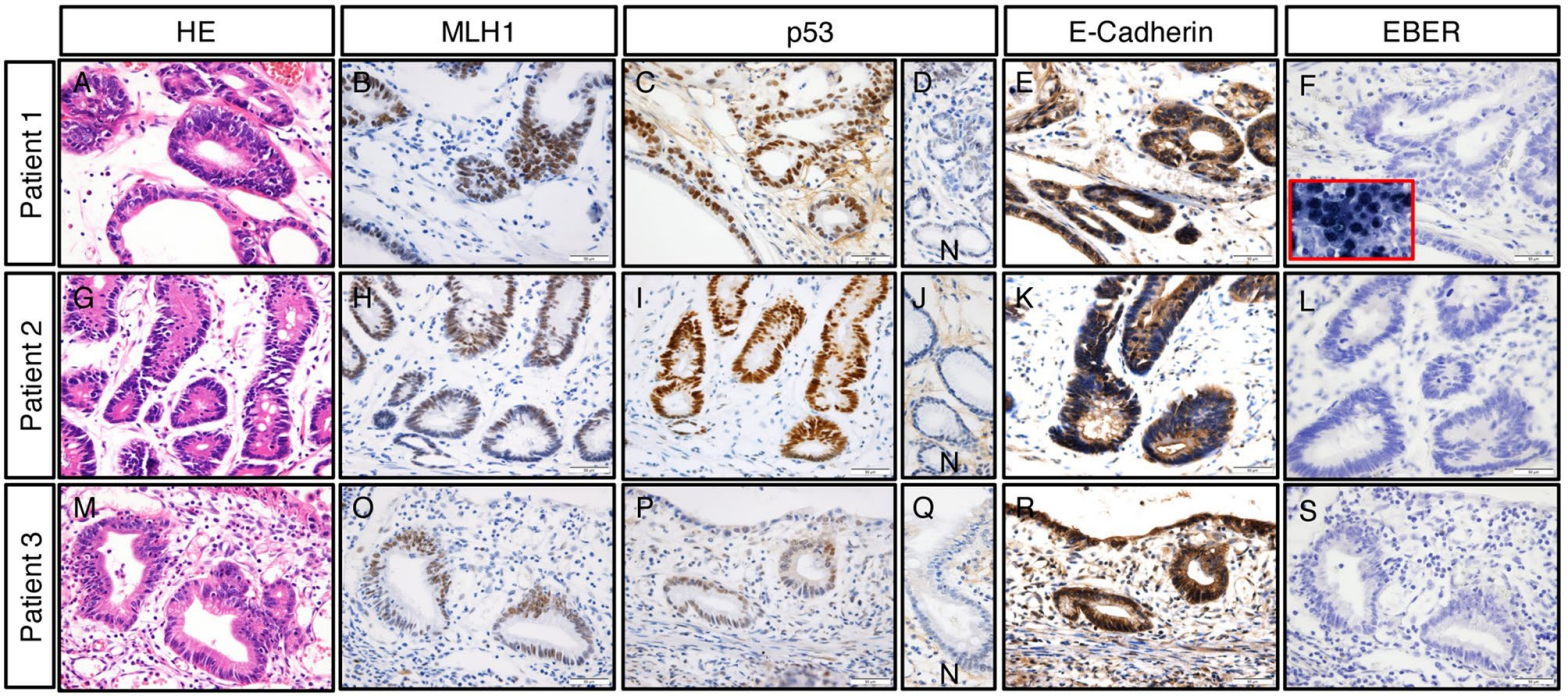
TCGA classification by immunohistochemistry and EBER for the three NHPH positive gastric cancer. (**A**,**G**,**M**) Hematoxylin and eosin staining for gastric cancer in Patients 1, 2, and 3, respectively. (**B**,**H**,**O**) MLH1 staining was positive for all three gastric cancers and no microsatellite instability was indicated. (**C**,**I**,**P**) p53 staining was positive and p53 was aberrantly expressed in all three gastric cancers. (**D**,**J**,**Q**) p53 staining in the non-cancerous part of gastric mucosa in each patient. P53 was not aberrantly expressed in normal regions. (**E**,**K**,**R**) E-cadherin was positive in all three cancers. (**F**,**L**,**S**) EBER in situ hybridization. Epstain-Barr virus was negative in all three stomachs. Inset in (**F**) shows positive control. (**N**) shows the surrounding non-cancerous parts. Taken together, all three gastric cancers were classified into the Chromosomal Instability (CIN) Type.

For specifying gastric cancer under infection of NHPH, we determined the TCGA classification of these three gastric cancers with immunohistochemistry for MLH-1, p53, and CDH-1, and EBER in situ hybridization. All three gastric cancer were well-differentiated adenocarcinoma, MLH-1 positive, p53-positive, CDH-1-positive, and EBER-negative, leading to classification as in the Chromosomal Instability (CIN) Type (Fig. 4).

## Discussion

We identified three NHPH positive patients with gastric cancer treated by endoscopic resection. Two of them showed co-infection with *H. pylori*, but the remaining patient had no evidence of *H. pylori* infection.

Gastric NHPH is reported to cause gastric MALT lymphoma and nodular gastritis with mild atrophy in the corpus^21^. However, the number of reports of gastric cancer associated with NHPH infection is very small^9–11^. Nogueira’s case was co-infected with *H. pylori* and no precise characteristics of gastric cancer and gastritis in this patient were reported^10^. Yang’s case did not check for *H. pylori* infection^11^. This patient had intestinal-type gastric cancer in the antrum, and mucosal inflammation and NHPH existed only in antrum. Morgner’s case did not examine *H. pylori* either^9^. The cancer in this patient was diffuse-type in the corpus, with moderate gastritis only in antrum. In the present report, *H. pylori* infection was analyzed with both serum IgG and PCR. Patient 1 was only positive for *H. pylori* by PCR and Patient 3 was only positive for *H. pylori* by serum IgG. The reason for the negativity of serum IgG in the Patient 1 is not clear, but the sensitivity of serum IgG for *H. pylori* infection is reported around 70% and this patient may be a false negative case. There are two possible reasons for the negativity of *H. pylori* PCR for Patient 3. One is PCR error for DNA extracted from paraffin sections, and the other is that this stomach was in the process of losing *H. pylori* due to extensive atrophy. However, the result of rapid urease test of Patient 3 was negative at the time of endoscopic treatment. The results became positive 3 months later and he was eradicated for *H. pylori*. From these facts, the *H. pylori* PCR results of Patient 3 may be false negative. In contrast, *H. pylori* was negative in Patient 2 by both serum IgG and PCR with minimal atrophy in gastric mucosa. *H. pylori* usually disappears in severe atrophy of the gastric mucosa after long term infection. However, Kimura-Takemoto classification of the gastric mucosa of Patient 2 was C-II, and thus natural disappearance of *H. pylori* is less likely. Patient 2 may really be negative for *H. pylori.* Therefore, in this patient, NHPH is speculated to have pathogenicity for gastric cancer. This is the first case of gastric cancer with NHPH infection, confirmed without infection of *H. pylori*.

We have previously shown that Trefoil Factor Family 3 (TFF3), a small peptide of 12–20 kD with a trefoil motif, which is secreted from various mucus-secreting cells, is an effective serum biomarker for gastric cancer both in Japan and in South Korea^12^. Although the mechanism of elevated serum TFF3 in cancer patients is not known, this biomarker showed a different behavior in Japanese and Korean gastric cancer patients. Korean early gastric cancer patients of Group A, negative for Pepsinogen test and low serum *H. pylori* IgG, have higher serum TFF3 than Group B, C, and D patients, who are positive for either the Pepsinogen test or *H. pylori* IgG. However, serum TFF3 in Japanese Group A gastric cancer patients was lower than in Japanese Group B, C, and D patients. From these results, we have speculated that causes for gastric cancer other than *H. pylori* infection exist in Korean people. Therefore, we investigated the presence of NHPH in gastric mucosa in 281 Korean early gastric cancer patients in this study and 3 gastric cancer patients with NHPH infection were found. In the literature, the NHPH positive rate in biopsy specimens from 5593 patients with upper gastrointestinal symptoms, analyzed by histology, was 0.17% in South Korea^22^. Although PCR would be more sensitive than histology, 3 out of 281 (1.1%) is much higher than 0.17%, and NHPH may have the possibility of causing gastric cancer.

We could not detect the precise strain of NHPH by PCR in these 3 patients. The sizes of PCR products for detecting the precise strain of NHPH are larger than PCR products for NHPH detection, and it may be the cause of PCR error for DNA extracted from paraffin sections. Patient 2 and Patient 3 were positive for *H. suis* by immunohistochemistry, the most prevalent gastric NHPH. Which strain of NHPH was present in Patient 1 is not known. In the literature, among 236 Japanese patients with gastric symptoms, not eradicated for *H. pylori*, and negative for *H. pylori* by rapid urease test, 49 patients (20.8%) were NHPH positive^5^. Among them, 20 patients were *H. suis* positive, 7 patients were *H. heilmannii* sensu stricto (*H. heilmannii* s. s.) positive, and 22 patients were not identified. Korean authors of this paper have found one patient infected with *H. heilmannii* s. s. in another Korean cohort of gastric cancer patients (to be published elsewhere) and this Patient 1 may also be infected with *H. heilmannii* s. s.

The origin of NHPH infection in these 3 patients is not known. The patients were not keeping pets, and were not working in animal handling^23,24^. The patients might have been infected with NHPH through food without proper cooking. *H. suis* is highly prevalent in pig stomach (60–80%)^20^. NHPH is lacking in a Cag pathogenicity island and would be less pathogenic than *H. pylori* . As to the virulence factor of *H. suis,* we would suggest HsvA protein, used in the immunohistochemical study, as one of the candidates because of its similarity to *H. pylori* autotransporter proteins ImaA, FaaA, and VlpC.

The presence of gastric cancer in the patient with NHPH single infection suggests that eradication of NHPH for preventing gastric cancer may be merited. NHPH can be eradicated by the same drugs used for *H. pylori*^6,13–15^. However, the urease activity of NHPH depends on the strain and NHPH single infection would show as rapid urease test negative, serum *H. pylori* IgG negative, stool *H. pylori* antigen negative, and urea breath test negative^6,15,16^. For the diagnosis of NHPH, it is important to consider the NHPH infection in patients without atrophic gastritis and negative for *H. pylori*. Diagnostic systems for NHPH infection are now under development.

In conclusion, we identified three patients with gastric cancer who were positive for NHPH, including one patient was without co-infection with *H. pylori*. NHPH could be one of the causes for gastric cancer in Korea and partially explain differences between serum TFF3 and ABCD classifications between Korea and Japan. NHPH eradication could be also important to prevent gastric carcinogenesis in this *H. pylori* eradication era.

## Supplementary Information

Supplementary Information.
