# Supplementary material for: Non-Helicobacter pylori Helicobacter (NHPH) positive gastric cancer
Source: Sci Rep. 2022 Mar 21;12:4811. doi: 10.1038/s41598-022-08962-y (PMC8938428; doi:10.1038/s41598-022-08962-y)
Supplement: Supplementary file 1 — Supplementary Information. [file 41598_2022_8962_MOESM1_ESM.pdf]

Supplemental Materials

Non-*Helicobacter pylori Helicobacter* (NHPH) positive gastric cancer

Tomohiko Yasuda, Hyun Seok Lee, Su Youn Nam, Hiroto Katoh, Yuko Ishibashi, Somay Yamagata Murayama, Hidenori Matsui, Hiroki Masuda, Emiko Rimbara, Nobuyuki Sakurazawa, Hideyuki Suzuki, Hiroshi Yoshida, Yasuyuki Seto, Shumpei Ishikawa, Seong Woo Jeon, Masahiko Nakamura, Sachiyo Nomura

Supplemental Figure S1 Specificity of Anti-*H. suis* Antibody

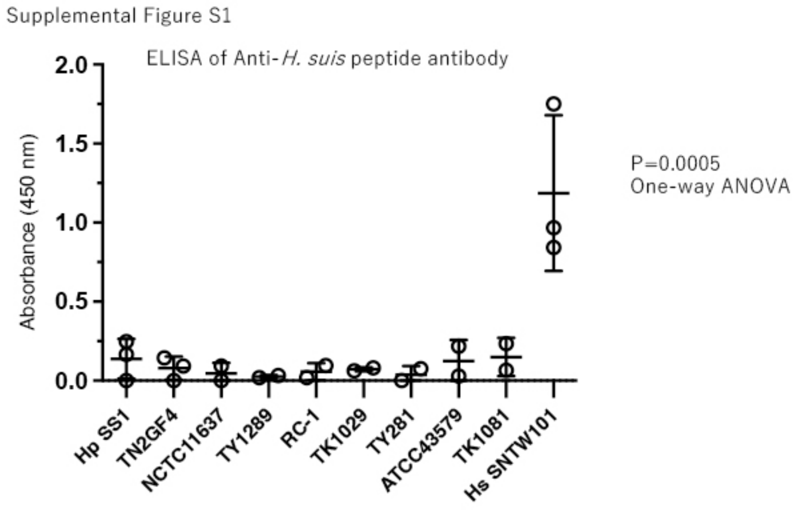

Supplemental Table S1

Rapid Urease Test Negative:0 Positive:1	pepsi I	pepsi II	I/II ratio	ABCD	H. pylori IgG (U/ml) (Normal <10)	initial (2011)H pylori IgG level by ELISA	initial H pylori IgG level positive/negative	Sex	Age
0	106.6	116.6	0.9	A	3	0.69	0	2	54
0	91	23.9	3.8	B	19	no result		1	62
0	44.1	11.7	3.8	B	24	0.93	Equivocal	2	78
0	73.7	15.5	4.8	A	3	no result		1	69
0	3.1	9.2	0.3	D	3	0.19	0	1	57
0	26.8	20.2	1.3	C	11	0.18	0	2	66
0	313.2	47.2	6.6	B	26	0.73	0	1	57
0	141.9	15	9.5	A	3	0.27	0	1	73
0	165.8	49.2	2.8	A	6	no result		1	66
1	30	13.2	2.3	C	36	1.68	1	1	69
0	39.1	6.7	5.8	A	5	0.44	0	1	77
0	130.6	16.3	8	A	3	0.22	0	2	67
0	52.7	10	5.3	A	3	no result		1	69
0	<2.5	5.4	<0.4	D	3	0.24	0	1	76
0	10	3	1.3	B	13	3.1	1	1	76
0	59.3	10.5	5.6	A	3	no result		1	58
0	48.7	8	6.1	A	3	0.08	0	1	66
0	19.1	6.7	2.9	D	3	0.46	0	1	70
0	8.2	8.3	1	C	18	no result		1	51
0	144.2	16	9	B	25	no result		1	74
0	56.8	8.3	6.8	A	5	0.97	Equivocal	1	69
0	46.8	5.1	9.2	A	4	0.72	0	1	68
0	31.4	5.3	5.9	A	4	1.00	Equivocal	1	48
1	21.3	19.6	1.1	C	12	0.90	Equivocal	1	76
1	46	18.2	2.5	C	19	3.82	1	1	57
0	118.5	52.8	2.2	B	33	4.60	1	2	76
0	<2.5	6.3	<0.3	D	3	1.00	1	1	86
0	3.8	6.5	0.6	D	3	0.59	0	1	71
0	75.2	25.1	2.99	D	3	0.21	0	1	67
1	88.1	29.7	2.96	B	21	1.82	1	1	66
0	33.9	5.6	6.1	A	7	0.86	Equivocal	2	57
0	14.2	19.4	0.7	D	7	2.51	1	1	77
1	17.5	13.9	1.3	D	8	0.15	0	2	74
1	57.9	19.6	2.95	C	23	0.93	Equivocal	1	58
0	49.7	8.6	5.8	B	11	no result		1	51
1	83.2	30.7	2.7	B	36	2.05	1	1	51
0	36.8	8.6	4.3	A	3	0.93	Equivocal	1	50
1	46.3	20	2.3	C	42	4.91	1	1	52
1	16.1	11.7	1.4	D	9	0.81	Equivocal	1	57
1	46.5	37	1.3	C	32	1.36	1	1	68
1	72.4	28.8	2.5	B	27	0.30	0	2	59
0	49	8.6	5.7	A	<3	no result		1	64
0	94.3	16.3	5.8	A	<3	0.58	0	1	73
0	36.1	7.4	4.9	A	<3	0.01	0	2	70
0	217.6	29	7.5	A	<3	0.48	0	2	76
0	63.9	10	6.4	A	3	no result		1	69
1	47.4	26.4	1.8	C	11	0.14	0	1	64
0	4.1	7.3	0.5	C	31	no result		1	62
1	105	35.4	2.96	B	20	0.42	0	1	61
1	84.1	46.4	1.8	C	10	no result		2	68
1	95.2	44.8	2.1	B	20	no result		2	72
1	114	65	1.8	B	32	no result		1	56
0	31	7.2	4.3	B	17	no result		1	42
0	52.6	10.5	5	A	7	1.25	1	1	63
1	11.1	6.7	1.6	C	21	no result		1	78
1	50.9	19.9	2.6	C	36	1.55	1	1	56
1	45.2	22.3	2	D	5	no result		1	73
0	56.5	23.4	2.4	D	<3	0.15	0	2	80
0	120.3	31.2	3.9	B	27	0.55	0	1	56
1	188.8	49.8	3.8	B	34	1.44	1	1	55
0	12.7	7.1	1.8	D	4	0.17	0	1	68
0	22.1	14.8	1.5	D	9	1.57	1	1	62
1	21.3	19.6	1.1	C	17	1.60	1	1	59
1	67	26.8	2.5	C	46	2.37	1	1	66
1	115.3	41.1	2.8	B	45	0.43	0	1	64
0	64.7	13	5	A	<3	1.42	1	1	60
1	111.1	38.1	2.9	B	61	no result		2	62
1	5	10.9	0.5	D	8	0.14	0	1	68
1	57.7	26.7	2.2	D	9	0.42	0	2	76
1	80.7	31.9	2.5	B	17	0.40	0	2	48
1	37.3	15.7	2.4	C	16	0.83	Equivocal	1	46
1	88.1	36	2.4	B	23	0.44	0	1	59
0	55.5	14.1	3.9	A	4	no result		1	52
0	39.5	8.3	4.7	B	47	no result		1	53
1	216.7	81.6	2.7	B	51	no result		1	70
1	11.5	24.7	0.5	C	32	no result		1	52
1	57	20.4	2.8	C	20	0.49	0	1	71
0	103.6	16.4	6.3	A	<3	0.08	0	1	63
1	56.1	19.1	2.9	C	62	0.99	Equivocal	1	60
0	148.8	25.1	5.9	B	20	no result		1	64
0	89.41	25.6	3.5	B	41	3.36	1	1	64
0	32.1	8.5	3.8	B	17	0.71	0	1	40
0	5	7.9	0.6	D	8	0.58	0	1	74
1	143.1	31.8	4.5	B	26	0.42	0	1	23
1	27.1	18.1	1.5	C	20	no result		1	74
1	13.3	17.1	0.8	C	38	no result		1	75
1	27.8	18.4	1.5	C	59	1.53	1	1	71
0	32.9	7.1	4.6	A	<3	no result		1	68
0	53.6	12.9	4.2	A	<3	0.32	0	1	79
1	137.6	45.2	3.04	B	15	0.62	0	1	55
0	87.6	23.8	3.7	B	60	2.71	1	1	60

1	78.1	28.9	2.7	B	48	0.95	Equivocal	1	60
0	196.6	24.9	7.9	A	3	0.07	0	1	63
1	132.4	61.5	2.2	B	58	2.99	1	2	64
1	63.8	26	2.5	D	8	no result		1	71
1	108.8	31.9	3.4	B	29	2.34	1	1	59
1	187	47.4	3.9	B	14	0.48	0	1	46
0	80.5	12	6.7	A	6	0.21	0	2	60
0	30.1	8.4	3.6	A	<3	0.53	0	1	61
1	67.9	48.6	1.4	C	38	1.12	1	2	56
1	82.8	39	2.1	B	64	1.58	1	2	77
1	33.6	16	2.1	C	25	no result		2	69
1	100.9	39.3	2.6	B	51	no result		1	57
1	71.7	32.9	2.2	B	21	0.13	0	1	72
0	5.5	21.5	0.3	D	7	2.19	1	2	58
0	39.3	12.2	3.2	A	<3	no result		2	73
0	128.9	43.4	2.97	B	45	1.71	1	2	70
0	5	5.4	0.9	C	44	0.05	0	1	71
0	153.7	22.5	6.8	A	<3	0.06	0	2	81
0	126.1	29.3	4.3	B	12	0.45	0	2	69
1	72.5	24.2	2.99	B	37	no result		2	79
0	74	45.1	1.6	B	46	no result		2	69
0	149.3	15	9.9	A	4	no result		1	71
0	44.9	24.8	1.8	C	39	0.69	0	2	63
0	63.1	22.2	2.8	C	28	no result		2	70
0	5	13.6	0.4	C	15	1.05	1	1	64
1	91.9	29.8	3.1	B	33	2.03	1	1	59
1	230.1	96.9	2.4	A	5	0.61	0	2	76
0	40.4	10.8	3.7	A	<3	0.51	0	2	66
1	6.6	18.2	0.4	C	10	0.24	0	1	59
1	47.8	22.7	2.1	C	30	0.90	Equivocal	2	71
1	51.3	17.3	2.96	C	13	0.77	Equivocal	1	57
0	106.8	21.9	4.9	A	<3	0.12	0	1	79
0	61.9	12.1	5.1	A	<3	0.11	0	1	65
0	62.4	17.1	3.7	A	3	0.45	0	1	68
1	43.1	21.2	2	C	14	negative	0	2	76
0	78.8	40.3	2	B	19	0.99	Equivocal	1	50
0	90.5	34.5	2.6	B	86	0.84	Equivocal	1	52
1	80.6	20.5	3.9	B	26	0.64	0	1	55
0	11.6	5.2	2.2	D	3	0.69	0	1	61
1	40.6	33.1	1.2	C	25	0.77	Equivocal	2	66
0	161.6	40.9	3.9	B	21	no result		1	64
0	24.8	8.4	2.95	D	3	0.80	Equivocal	1	78
0	50.1	9.7	5.2	A	6	0.94	Equivocal	2	48
1	76.8	24.7	3.1	B	12	0.29	0	1	52
0	40.9	9.7	4.2	A	<3	0.23	0	2	72
1	236.4	75.6	3.1	B	23	0.98	Equivocal	2	69
1	71.6	32.8	2.2	B	72	3.18	1	1	57
0	22.9	10	2.3	D	3	0.26	0	1	71
0	45	29.4	1.5	C	87	2.70	1	1	76
1	54.8	19.4	2.8	C	14	1.07	1	2	56
1	111.3	52.2	2.1	B	10	no result		2	72
1	194.5	49	4	B	10	0.73	0	1	57
1	81.3	39.1	2.1	B	27	1.2	1	1	59
0	36.6	23.3	1.6	C	20	1.22	1	1	67
0	24.2	20.9	1.2	C	19	4.23	1	1	78
0	105.7	15.1	7	A	5	1.59	1	1	66
1	43.9	15.9	2.8	D	4	0.03	0	1	71
1	69.1	18.3	3.8	C	18	53.6	1	2	49
0	123	25.3	4.9	A	<3	0.08	0	1	63
0	63.5	17.4	3.7	A	<3	2.76	1	1	51
1	106.2	27.7	3.8	B	10	28.8	1	1	64
0	15.3	19	0.8	D	7	24.5	1	1	77
0	91.4	21.5	4.2	A	8	no result		1	53
1	35.4	14.5	2.4	C	18	0.84	Equivocal	2	59
0	65.8	15.5	4.3	A	4	0.41	0	1	68
1	57.4	15.4	3.7	B	18	negative	0	1	53
0	43.2	16.2	2.7	C	52	1.13	1	2	53
0	13.7	8.2	1.7	C	12	0.79	Equivocal	1	55
0	45	15.9	2.8	C	41	1.39	1	1	63
0	38.4	9.1	4.2	A	<3	0.25	0	2	60
0	118.6	29.6	4	B	21	0.49	0	1	60
0	93.1	14.6	6.4	A	<3	negative	0	1	64
0	92.1	25	3.7	B	24	0.94	Equivocal	1	61
1	87.9	30.9	2.8	B	13	179.6	1	1	53
0	7.1	4.8	1.5	C	12	no result		1	66
0	43.3	7.7	5.6	A	6	negative	0	1	54
0	106.3	24.3	4.4	B	39	positive	1	1	75
0	58.8	11.7	5	B	24	negative	0	1	58
0	85.5	33.4	2.6	B	61	4.41	1	1	77
0	100	14.2	7.1	B	12	no result		1	51
1	37.7	11.5	3.3	A	9	<5	0	1	48
0	224	39.6	5.7	A	<3	<5	0	2	60
0	23.5	9.8	2.4	C	24	negative	0	1	68
1	91.6	35.3	2.6	B	35	64.8	1	1	64
0	22.6	22.8	1	C	21	140.9	1	1	66
1	71.2	22	3.2	B	32	6.7	0	1	61
0	6.7	13.6	0.5	D	5	0.1	0	1	72
0	81.9	38.6	2.1	B	28	138.5	1	1	48
0	119.7	36.4	3.3	B	14	24.2	1	1	70
0	101.6	44.1	2.3	B	59	3.88	1	1	64
1	114.1	55.4	2.1	B	53	20.1	1	1	55
1	78.6	20.8	3.8	B	35	7.8	0	1	56
0	56.2	11.5	4.9	A	3	9.7	Equivocal	1	66
1	106.8	50.2	2.1	B	19	no result		2	66
1	110.8	37.5	2.95	B	10	7.6	0	1	72
0	24.8	36.6	0.9	D	7	no result		1	71
1	41.4	12.7	3.3	B	18	6.7	0	2	55
0	73.3	26	2.8	B	19	45.4	1	1	64

0	205	42.3	4.8	A	3	no result		1	71
0	47.6	29.6	1.6	C	41	84.4	1	1	58
1	37.4	27.1	1.4	C	13	17.8	1	2	70
0	5	9	0.6	D	8	88	1	1	68
0	112.4	14	8	B	22	76.6	1	1	63
1	19.4	14.3	1.4	C	50	87.4	1	2	60
1	145.9	38.6	3.8	B	21	7.1	0	1	64
0	43	16.2	2.7	C	41	no result		1	57
0	115	20.2	5.7	A	4	20.8	1	1	69
1	237.2	65.8	3.6	A	<3	7.3	0	1	48
0	15.2	10	1.5	D	8	40.7	1	1	52
0	47.8	8.4	5.7	A	8	6.7	0	2	64
1	120.4	39.4	3.1	B	39	55	1	1	68
0	5	10	0.5	C	41	no result		1	74
1	20.9	17	1.2	C	54	8	Equivocal	1	74
1	78.1	31.9	2.4	B	39	7.5	0	1	58
0	14.2	6.7	2.1	C	13	no result		1	54
0	77	48.2	1.6	B	31	no result		2	80
0	43.2	30.7	1.4	C	50	169.6	1	1	66
0	129.7	70.8	1.8	B	12	80.5	1	1	55
0	49.8	7.9	6.3	A	<3	6.2	0	1	54
0	55.9	15.1	3.7	A	<3	6.1	0	1	64
0	19.2	23.6	0.8	D	9	23.7	1	2	54
0	5	6.4	0.8	D	3	no result		1	66
0	12.2	5.3	2.3	C	14	3.53	1	1	65
0	5.5	4.9	1.1	D	4	5.6	0	2	72
1	80.5	20.2	4	B	13	10	Equivocal	1	50
0	21.8	22.5	1	C	15	137.6	1	1	68
0	14.1	11	1.3	D	3	10.3	Equivocal	2	64
0	112.8	37.8	2.98	B	38	153.7	1	1	58
0	45.1	30	1.5	C	72	131	1	1	65
0	44.4	25.8	1.7	D	4	29.1	1	1	68
1	42.8	37.5	1.1	D	7	5.8	0	1	66
0	69.1	30.4	2.3	C	14	no result		1	58
0	21.3	28.6	0.7	C	18	39.6	1	2	70
0	6.8	6.3	1.1	C	11	143.2	1	1	62
0	43.9	21.9	2	C	14	6.5	0	1	64
0	73.2	12.1	6	B	38	no result		1	34
1	64.3	17.8	3.6	B	19	5.4	0	2	55
0	101.2	15	6.7	A	4	7.9	0	1	53
0	50.1	14	3.6	A	7	no result		1	57
1	37.8	17.8	2.1	C	15	92.7	1	1	79
1	23.5	11.7	2	C	12	13.9	1	2	72
0	30.5	6	5.1	A	3	25.2	1	1	57
1	130.2	35.6	3.7	B	41	36.5	1	1	61
0	113.6	39.7	2.9	B	19	63.4	1	2	51
1	38.6	19.7	2	C	23	105	1	1	57
1	37.1	18.9	2	C	14	83.9	1	1	58
0	133.2	42.4	3.1	A	<3	no result		1	64
1	79.7	20.1	4	B	10	17.6	0	1	68
1	83	34.4	2.4	B	24	188.9	1	1	77
1	78.7	21.3	3.7	B	55	135.9	1	1	54
1	8.8	10.1	0.9	C	46	no result		2	72
1	41.4	21.9	1.9	D	3	2.79	1	1	64
1	30.5	22.8	1.3	D	9	0.51	0	1	74
1	41.8	19.4	2.2	C	15	no result		1	62
1	153.7	98.6	1.5	B	24	0.98	Equivocal	2	68
1	98	32.8	2.98	B	46	1.46	1	1	60
1	33.8	16.4	2.1	C	13	3.64	1	1	62
1	75.4	29.4	2.6	B	11	0.53	0	2	67
1	133.7	42.4	3.2	B		no result		1	49
1	31.9	37.2	0.9	C	15	0.47	0	2	63
1	71.2	18.7	3.8	B	29	1.4	1	1	76
1	14.6	12.8	1.1	C	35	4.71	1	1	68
1	141.3	45	3.1	B	40	no result		1	63
1	51.3	23	2.2	C	21	0.8	Equivocal	2	45
1	85.6	40.2	2.1	B	36	2.55	1	1	62
1	9	10.3	0.9	C	10	no result		1	69
1	64.1	19.5	3.3	B	18	0.74	0	1	66
1	69.7	25.9	2.7	C	24	0.85	Equivocal	1	45
1	61.2	20.7	2.95	C	31	0.98	Equivocal	1	59
1	59.2	21.4	2.8	D	9	0.56	0	1	67
1	53.2	18.5	2.9	B	18	1.04	1	2	71
1	57.6	15.5	3.7	B	16	0.11	0	2	67
1	100.8	31.3	3.2	B	18	0.27	0	2	74
1	41	20.1	2	C	11	0.42	0	2	75
1	83.8	16.5	5.1	A	3	no result		2	78
1	86.9	29.3	2.96	B	44	4.27	1	1	59
1	6.3	7.4	0.9	C	25	1.41	1	1	72
1	58.7	18.7	3.1	B	17	0.19	1	1	74
1	87.9	21.4	4.1	B	15	1.31	1	1	56
1	69.2	15.4	4.5	B	39	2.06	1	1	62
1	59.1	13.8	4.3	B	30	1.09	1	1	71
1	60.8	17.4	3.5	B	37	1.55	1	1	58
1	151.8	33.5	4.5	B	32	2.81	1	1	66
1	161.5	43	3.8	B	17	1.19	1	1	46
1	95.2	27.2	3.5	A	7	no result		1	46
1	59.6	19.8	3.01	C	17	0.46	0	1	72
1	68.3	17.5	3.9	A	9	no result		1	64
1	116.8	33.8	3.5	B	20	1.43	1	1	61
1	120.1	36.4	3.3	B	26	1.9	1	1	59
1	119.1	35.1	3.4	B	54	no result		1	42

The green highlighted are NHPH positive patients
